# Successful long-term results (> 5 years) of superselective transarterial chemoembolization in symptomatic/enlarging liver hemangiomas: a paradigm shift at a hepatopancreatobiliary surgery unit

**DOI:** 10.3389/fmed.2026.1699596

**Published:** 2026-01-27

**Authors:** İlgin Özden, Arzu Poyanlı, Cem İbiş, Bülent Acunaş

**Affiliations:** 1 Hepatopancreatobiliary Surgery Unit, Department of General Surgery, Istanbul Faculty of Medicine, Istanbul University, Istanbul, Turkey; 2 Department of Radiology, Istanbul Faculty of Medicine, Istanbul University, Istanbul, Turkey

**Keywords:** bleomycin, enlarging, hemangioma, lipiodol, superselective, transarterial chemoembolization

## Abstract

**Background:**

The favorable experience with superselective transarterial chemoembolization (TACE) using lipiodol-bleomycin may lead some hepatopancreatobiliary surgery centers to offer it as first-line treatment for symptomatic/enlarging hemangiomas.

**Patients and methods:**

The charts of 56 patients treated at a university hospital between 2012 and 2018 were reviewed. The results were reported as median (range).

**Results:**

The indication was abdominal pain in 46 patients (concomitant enlargement in 12, enlargement and fever in 1), asymptomatic enlargement in 8 and possibility of adverse hemodynamic consequences in two. A single session was planned for 48 patients and two-sessions in 8; in addition, four patients required 2 (*n*: 2) or 3 (*n*: 2) sessions for symptom control. Six patients (11%), experienced post-embolization syndrome lasting longer than one week. Lesion volume decreased from 586 (147–8,435) cm^3^ to 332 (24–4,710) cm^3^ in 4 (2–8) months after the first session [*p* < 0.01; 46% (5–92) regression]. In the 8 patients who underwent two planned sessions, lesion volume decreased from 1,454 (441–8,435) cm^3^ to 661 (159–3,716) cm^3^, 5 (3–7) months after the second session [62% (37–78) regression]. Shrinkage in the 95%–99% range was observed in 13 (25%) of the 51 patients who were followed at least one year. Thirty-four (73%) of the 46 symptomatic patients reported resolution/marked amelioration of symptoms. No late complications were observed in 41 patients (73%) followed for at least 5 years; progressive regression was observed in 36 (88%) cases; in two patients (5%), initial regression was followed by regrowth.

**Conclusion:**

TACE is a successful first-line treatment for patients with symptomatic/enlarging hemangiomas. Better assessment of the quality of life in symptomatic patients and different definitions of success in cases with symptomatic and asymptomatic progressive enlargement are required.

## Introduction

Treatment is indicated in a very small subset of liver hemangiomas—the most common liver tumor– for severe abdominal pain, enlargement, diagnostic uncertainty and rare conditions such as rupture and the Kasabach–Merrit syndrome. Surgery has long been the traditional intervention method (1). On the other hand, remarkable expansion of knowledge on the treatment of symptomatic/enlarging liver hemangiomas with transarterial chemoembolization (TACE) using the lipiodol-bleomycin has been achieved in the last two decades: regression has been realized in 90% of the cases and symptom control in 63%–100%; severe complications have been generally rare (3%) [reviewed in Furumaya et al. (2) and Torkian et al. (3)]. Data on a significant number of patients followed for at least 3 years (some longer than 15 years) have been reported (4, 5). This favorable benefit-risk profile has led some authors to even consider expansion of treatment indications (6, 7). Despite these advances, many surgical departments still offer surgery as the primary treatment method to a significant number of patients with symptomatic/enlarging hemangiomas (8–11). To the best of our information, there is only one report on the experience of a hepatopancreatobiliary surgery unit which has considered TACE as first-line treatment (12). However, Liu et al. (12) stated that the results were unsatisfactory due to insufficient efficacy and unacceptably high morbidity and concluded that “Surgical treatment should be the preferred treatment option for patients with giant liver hemangioma which caused symptom.”

In contrast, the experience at our center has been much more favorable (13). The operation of choice in liver hemangiomas—enucleation– had been first reported in the international literature from our department (14) with favorable long-term results (15). The search for safer alternatives in the treatment of a benign lesion was stimulated by the referral of patients who would probably require a liver transplantation as a primary or salvage procedure (13); eventually, our approach has evolved to offering superselective TACE as the primary treatment in the majority of patients with symptomatic/enlarging hemangiomas based on the experience in 25 patients and the published literature (2, 3, 5, 13).

In this report, we communicate our favorable long-term (> 5 years) results in 56 patients to provide a “surgical perspective” from a hospital that can provide enucleation, resection and transplantation (deceased or living donor) but also maintains a close collaboration between the surgery and the interventional radiology departments.

## Patients and methods

The charts of 56 patients who underwent TACE for large symptomatic/enlarging hemangiomas between 2012 and 2018 were reviewed. This retrospective observational study was conducted according to the 1964 Helsinki Declaration and its amendments. Ethical approval was given by the institutional review board (File 2024/393).

The detailed approach for the evaluation of treatment indication, the TACE technique and periprocedural medical management scheme have been reported previously (13). Briefly, the diagnosis of hemangioma was made with dynamic contrast-enhanced magnetic resonance imaging (MRI). Other possible causes of abdominal pain were investigated by ultrasonography of the gallbladder, esophagogastroduodenoscopy, colonoscopy, computed tomography of the thorax and cardiologic examinations as indicated by complaints of the individual patients. Standard biochemistry tests, complete blood count and tumor marker measurements (CEA, CA 19–9 and AFP) were performed. Patients with a suspicion of malignancy underwent surgery. TACE was proposed as the primary treatment to patients with symptomatic/enlarging lesions. Asymptomatic patients were usually offered follow up only; however, TACE was considered for lesions showing obvious enlargement beyond 10 cm and beginning to abut major vascular structures (13).

The superselective TACE technique (13): First, a splenic or superior mesenteric artery injection was performed with a 4F glide Simmonds catheter in order to depict the arterial and portal anatomy. Then, the hepatic artery was selectively catheterized in order to identify the feeder arteries as well as to exclude arterioportal shunts. Feeding segmental arteries were catheterized with a 2.4 or 2.7F microcatheter. The lipiodol-bleomycin mixture (15 mg of bleomycin was dissolved in 5 mL of non-ionic contrast agent; this solution was mixed to homogeneity with 10 mL of lipiodol) was administered slowly through the microcatheter under fluoroscopic guidance. The injection was stopped when stagnation was achieved or the total volume was administered. The two most critical points are to use of microcatheters to embolize feeder arteries superselectively to avoid damage to the adjacent parenchyma and bile ducts and limit the maximum doses of bleomycin (15 mg) and lipiodol (10 mL) per session.

In the initial years, two sessions were planned in patients with very large lesions, particularly those receiving feeder vessels from both the right and left hepatic arteries (staged treatment) (13). However, the progressive nature of volume reduction over time (please see “Results”) led us to convert to an “on-demand” instead of an upfront approach, i.e., additional sessions were performed only in patients with persistent symptoms.

Complications were reported according to the SIR classification (16).

Symptom evaluation was made by asking patients to report their symptom status as follows: (1) complete resolution (2) marked amelioration (3) amelioration followed by aggravation (4) persistence. This scheme which had been used previously in our department in the evaluation of long-term results of surgery (15) and embolization (13) was employed again for consistency. Lesion volume was estimated by multiplying the largest three dimensions of the lesion and dividing by 2 (instead of 6/π). In patients in whom two neighboring lesions were embolized, the total volume was reported.

In patients who reported returning to normal life in the outpatient visit 7–10 days after the TACE, a non-contrast-enhanced MRI [computed tomography (CT) in some instances] examination and blood tests were recommended at 4 months and at 12 months. Annual follow up with MRI (usually non-contrast-enhanced) and biochemical examinations was preferred afterward in patients who had symptom improvement. Patients who lived in distant locations and did not wish to travel were interviewed on the telephone; they had their examinations done locally and sent the results to our center.

Data were reported as median (range) and evaluated by the Mann–Whitney U-test and Wilcoxon’s paired test as appropriate. Differences with *p*-values less than 0.05 were considered as statistically significant.

## Results

Between 2012 and 2018, liver hemangioma was one of the primary diagnoses in 879 patients referred to our unit, after excluding cases with previous treatment for any malignancy (except for well-differentiated thyroid carcinoma), history of liver surgery for any indication and cirrhosis. The approaches taken and the indications for surgical intervention (enucleation or hepatectomy) were summarized in Table 1. Except for diagnostic uncertainty and suspected limited rupture, surgery was performed very rarely, and only for individualized indications.

**TABLE 1:** Treatment approach to liver hemangiomas (*n*: 879).

Approach method	Number of patients
Follow up only	805
TACE	58 (attempted)
Surgical intervention	16
Diagnostic uncertainty	9
Very large (15, 25 and 30 cm) mostly exophytic lesion	3
Patient’s preference for a mostly exophytic lesion (8 and 10 cm)	2
Access to the hepatoduodenal ligament	1*
Suspected limited rupture	1

*A 20 cm-hemangioma prevented access to the hepatoduodenal ligament in a patient with multiple common bile duct stones which could not be treated by ERCP due to cannulation failure.

The TACE procedure was attempted in 58 patients and accomplished in 56: superselective catheterization was not possible in one patient; allergic reaction developed after premedication in a 52-year-old patient who had undergone surgery for other reasons; subsequent testing revealed hypersensitivity to meperidine. The technical success rate was 56/57 (98%).

**FIGURE 1:**
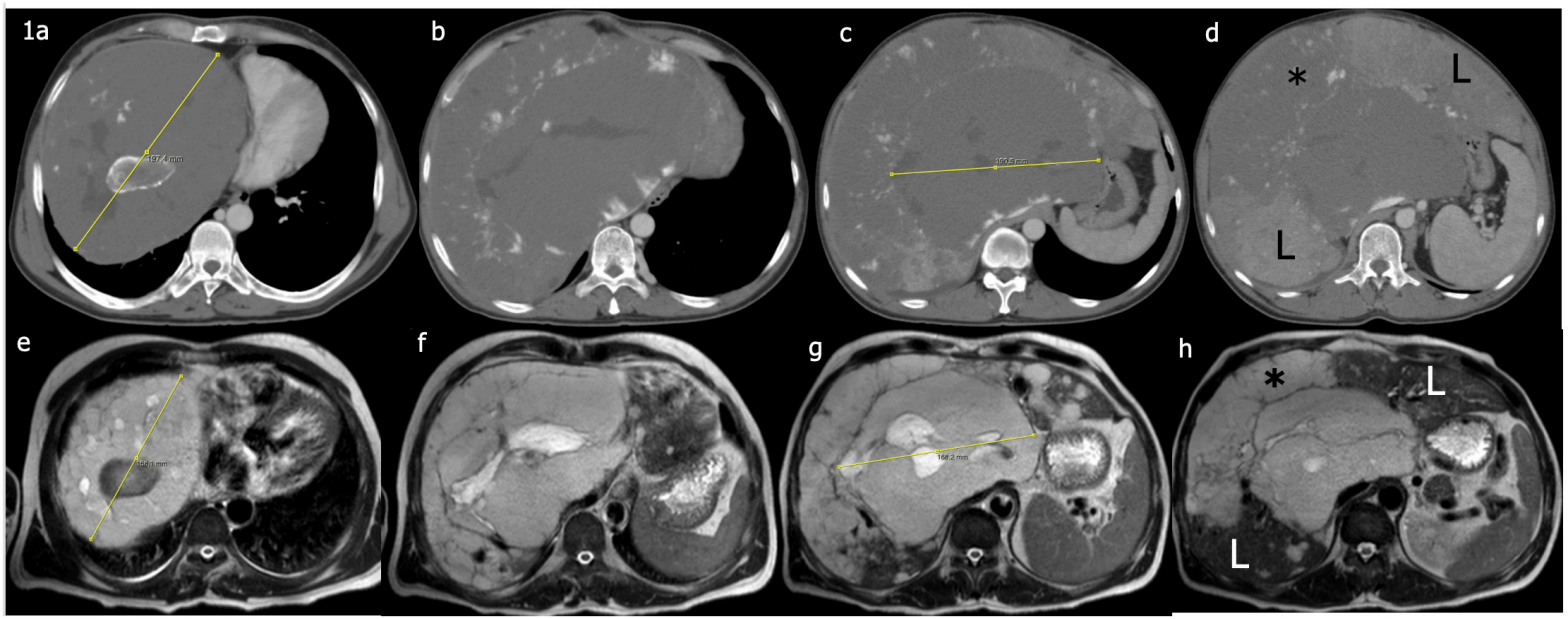
**(a–d)** Giant hemangioma arising from the caudate lobe, compressing the heart and segregating the right and left lobe parenchyma (L); hemangiomatous changes were evident in the adjacent areas (*). **(e–h)** MRI taken 4 months after TACE. Marked regression is evident. The asymptomatic patient refused further radiologic investigations.

**FIGURE 2:**
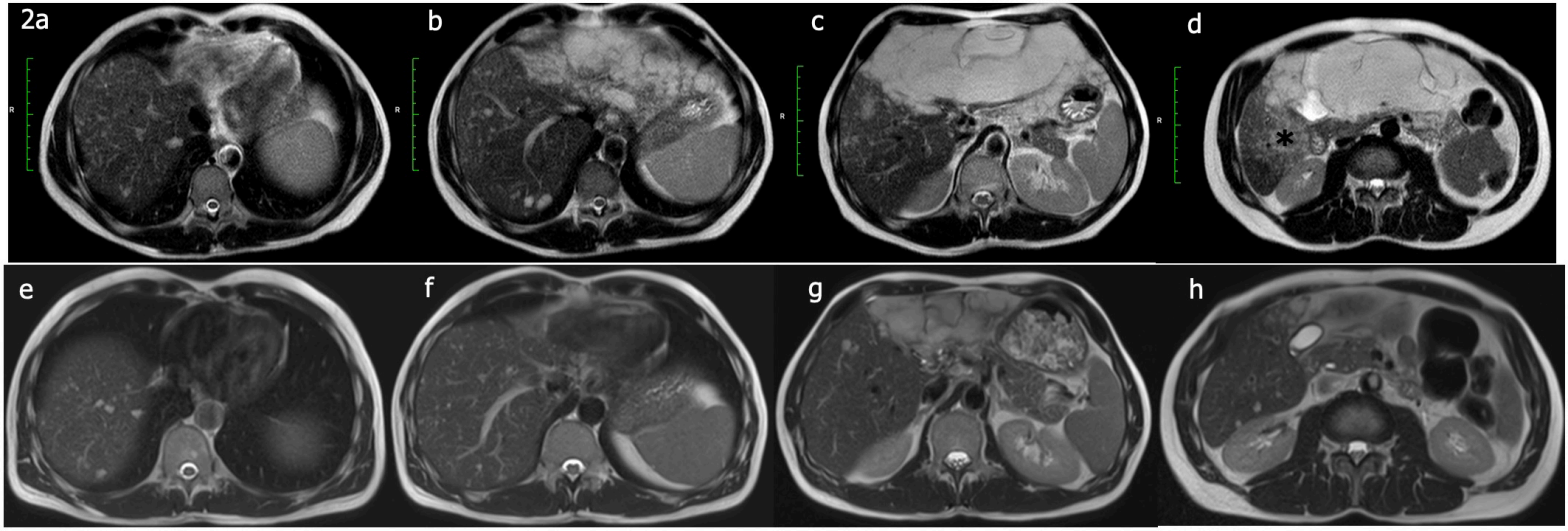
**(a–d)** Giant hemangioma extending from the left lobe to segment 6; hemangiomatous changes were evident in the adjacent areas (*). **(e–h)** MRI taken 4 years after a single TACE session. Marked regression is evident.

Forty-seven patients were women, 9 were men; median age was 48 (32–63). Twenty-four patients had a single hemangioma, 11 had two and 21 had three or more hemangiomas. The leading indication for treatment was abdominal pain (46 patients); 12 of these patients had concomitant enlargement, one had enlargement and fever. Eight patients had asymptomatic/mildly symptomatic enlargement. The possibility of adverse hemodynamic consequences in the long-term was the indication in two asymptomatic patients who had a giant hemangioma and concomitant hemangiomatous change in the adjacent parenchyma (Figures 1, 2).

Three patients had undergone unsuccessful embolization (no symptom control or size regression) at other institutions. Two patients had undergone enucleation for liver hemangiomas elsewhere but had “recurrent” lesions in the same locations.

Three patients had cholecystolithiasis and one had a 6.5 cm cystadenoma (the hemangioma was 16 cm); hemangioma treatment was given priority. Two patients had peptic ulcer which was treated medically; persistence of complaints led to the decision to perform TACE. One patient with a 5,946 cm^3^-hemangioma had been on the waiting list of another transplant center for 5 years.

In 50 patients, minor post-procedural complaints and liver function test abnormalities were observed (Class B). One patient developed a transient allergic rash that required steroid treatment (Class B). Six patients (11%) experienced a post-embolization syndrome that persisted for longer than one week (Class C–D). The condition resolved with analgesics and antibiotics in all cases but required hospitalization longer than 48 h. Prolonged postembolization syndrome was not related to lesion size: the lesion volumes of the 6 patients were 237, 265,360, 518, 586 and 829 cm^3^. One of these patients (lesion volume 586 cm^3^) required two rehospitalizations (total duration 16 days) for abdominal pain requiring parenteral analgesics and antibiotics and returned to a near-normal quality of life in 6 weeks. No patient experienced significant liver function impairment, infection (e.g., cholecystitis, cholangitis or abscess formation) or vascular complication.

The lesion volume decreased from 586 (147–8,435) cm^3^ to 332 (24–4,710) cm^3^ in 4 (2–8) months after the first session [*p* < 0.01; 46% (5–92) regression] in 49 patients (Table 2). Complete data was unavailable in 7 patients because the patients did not return from their hometown or country for early follow up or underwent the initial examination at another hospital and the CDs were unavailable for the purposes of this study.

**TABLE 2:** Changes in lesion volume after a single TACE session (*n*: 49).

Pre-TACE	Post-TACE
586 (147–8,435) cm^3^	332 (24–4,710) cm^3^

Lesion size decreased significantly (*p* < 0.01) by 46% (5–92) after 4 (2–8) months.

In the 8 patients who underwent two planned sessions, lesion volume decreased from 1,454 (441–8,435) cm^3^ to 942 (398–4,710) cm^3^ in 4 (2–6) months after the first session [34% (8–52) regression; *p* < 0.01]; the second session was performed 6 (3–8) months later and the lesion volume decreased further to 661 (159–3,716) cm^3^ in 5 (3–7) months [62% (37–78) regression; *p* < 0.01] (Table 3).

**TABLE 3:** Changes in lesion volume after two planned TACE sessions (*n*: 8).

Pre-TACE	Post-TACE1*	Post-TACE2**
1,454 (441–8,435) cm^3^	942 (398–4,710) cm^3^	661 (159–3,716) cm^3^

*4 (2–6) months after the first session; 34% (8–52) regression; *p* < 0.01. **5 (3–7) months after the second session; 62% (37–78) regression, compared to baseline; *p* < 0.01.

**FIGURE 3:**
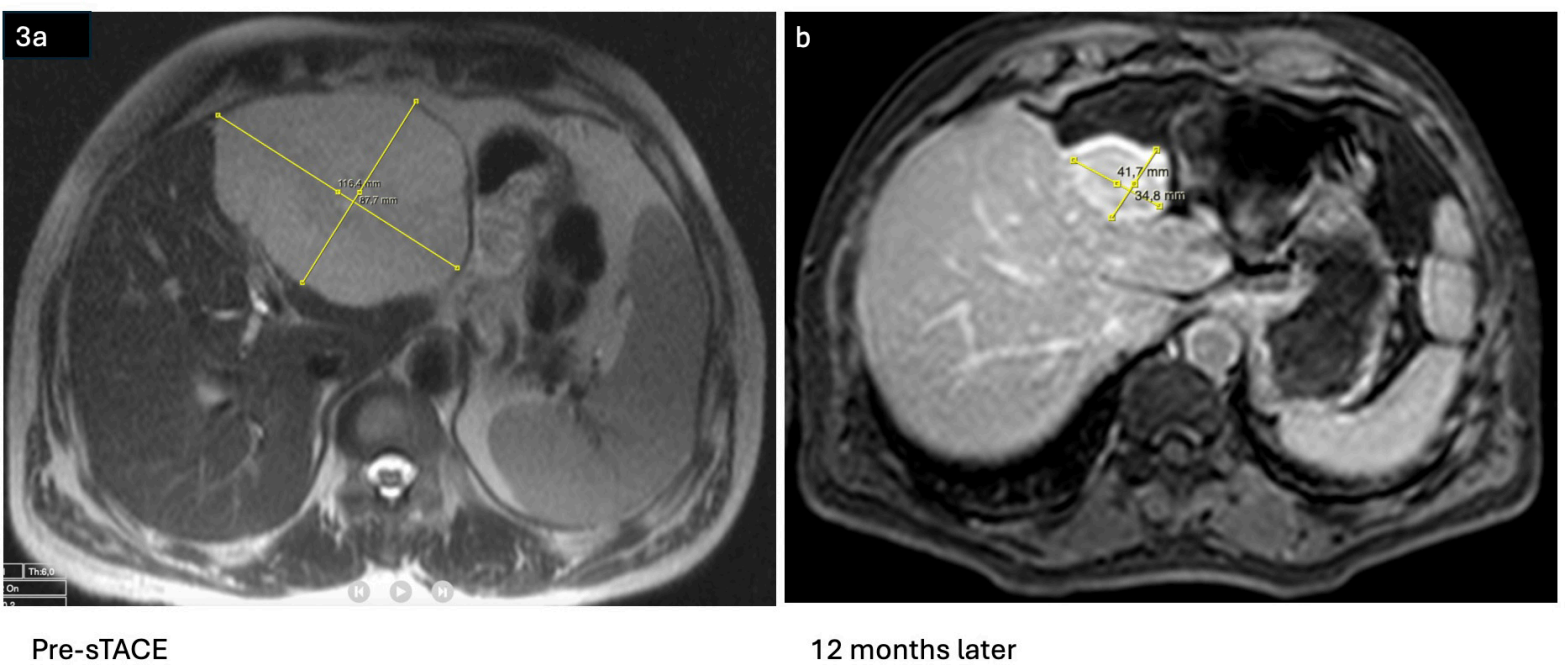
**(a,b)** Marked regression in one year after a single TACE session.

Regression in the 95%–99% range was observed in 13 (25%) of the 51 patients who were followed at least one year (Figure 3).

Thirty-four (73%) of the 46 symptomatic patients reported complete resolution/marked amelioration of symptoms. This required additional TACE sessions in four patients (eventual success in three). Three of the remaining 12 patients with unsatisfactory symptom control developed *de novo* cholecystolithiasis during follow up and unsatisfactory symptom control led to cholecystectomy; one of these had a cystadenoma which was resected as well. Two of these patients became asymptomatic; it is possible that original symptoms were due a gallbladder stone missed in the initial investigations. None of the three patients with cholecystolithiasis at baseline required a cholecystectomy. No other cause of pain could be identified in the remaining patients none of whom wanted to undergo surgery for the residual hemangioma.

The patient who presented with fever was a 40-year-old woman who complained of right upper quadrant pain and intermittent fever of 15 days duration. She had been followed for an asymptomatic liver hemangioma for 4 years at other institutions. Standard laboratory examination showed no abnormalities except for an increased CRP level (56 mg/L; ≤ 5 mg/L). MRI performed 4 years ago showed a 12.4 cm-hemangioma (approximately 595 cm^3^) in the right lobe; the current MRI showed that the lesion had enlarged to 13.3 cm (approximately 712 cm^3^) and an 8 cm sclerotic area had developed in the center. Pain and fever disappeared completely in 2 weeks after TACE and did not recur in 5 years of follow up during which the lesion volume decreased progressively to 38 cm^3^.

**FIGURE 4:**
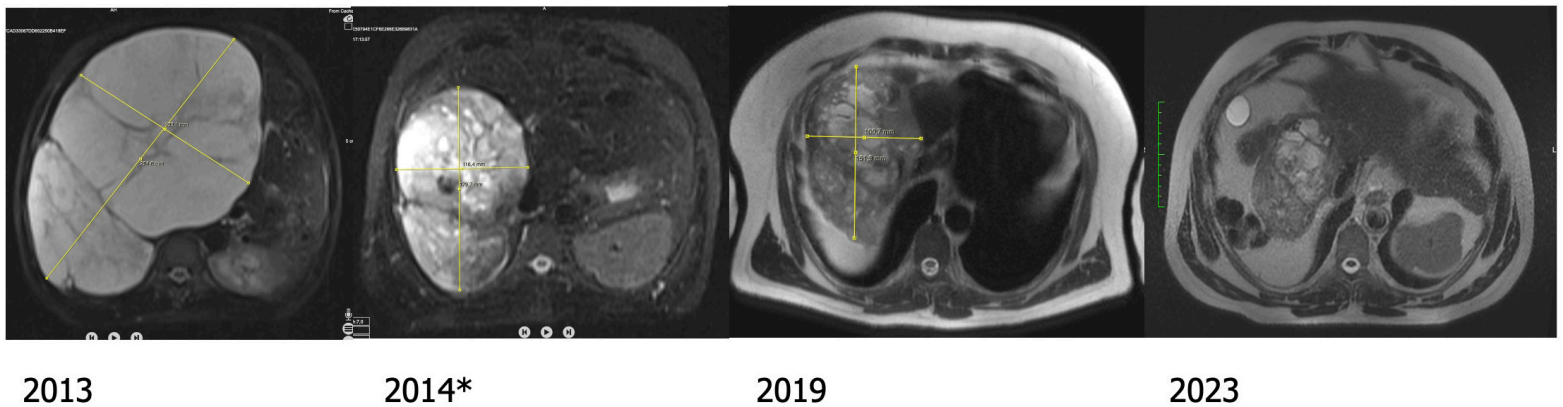
The patient underwent two planned TACE sessions. The MRI taken 6 months after the second session* shows significant volume regression which progressed even after 5 years.

No late complications were observed in 41 patients (73%) who were followed for at least 5 years; progressive regression was observed in 36 (88%) cases (Figure 4). In two patients (5%), initial regression was followed by gradual regrowth to the original size; one had been treated for symptoms and the other for enlargement. They chose conservative follow up.

## Discussion

The root-cause of the difficulty in managing liver hemangiomas is that a very small percentage of the patients with this common finding (1%–20%) (1) require extensive investigation or treatment after proper baseline assessment. However, deficiencies in the management of that small subset may have grave consequences: e.g., misdiagnosis of malignancy as a hemangioma (17) or conservative follow up in a patient with an enlarging hemangioma and concomitant hemangiomatosis, who eventually requires a liver transplant (18, 19). In order to allocate enough time and attention to hemangioma patients without infringing on the rights of the patients with malignancies and semi-urgent conditions at our hospital, a separate time interval for outpatient appointments was designated, starting from 2012 (20). An intervention was considered necessary in only 8.4% of the patients diagnosed during the study period and TACE has been the treatment of choice in symptomatic/enlarging lesions.

Size regression is the most objective outcome parameter for TACE. Decrease in hemangioma size has been reported in 90% of the cases (2, 3). In many studies, the change in the largest dimension is reported (2, 3). While this is acceptable for the presence or absence of regression, it may not be accurate enough, at least in some cases, in the assessment of the degree of the response. As stated in our previous report (13), the volumes of two hemangiomas with largest dimensions of 29 and 30 cm may vary as much as 5,173 vs. 8,435 cm^3^ and two hemangiomas measuring 11 cm as much as 233 vs. 636 cm^3^. The size regression after TACE with lipiodol-bleomycin is usually progressive, as reported previously (5, 13, 21). Since it may continue even after 5 years (Figure 4), it may be advisable to wait for at least one year instead of planning two sessions upfront.

Another aspect to consider is the degree of regression which should be considered a “success.” Radiologic success (5) and effectiveness (21) have been defined as at least 50% reduction in volume. While this is logical from an imaging point of view, further refinement is necessary from a clinical perspective. Different criteria should be applied to patients according to the primary indication for treatment. First, if the indication is progressive enlargement, a lesser degree of regression or even prevention of enlargement may be considered a favorable outcome. For example, in the present series, one of the two patients who experienced initial regression followed by regrowth had been treated for asymptomatic enlargement. The outcome of this patient may be considered a “radiologic failure” but also a “clinical success.” A second issue of divergence between the two evaluations, is a less-than-50% size regression in a patient who was treated for symptoms and reported satisfaction with treatment: again a “radiologic failure” but a “clinical success.”

Symptom control is the most frequently cited but the least clearly defined indication for intervention in liver hemangioma cases. The wide variation of the frequencies reported (between 63% and 100%) reflects that there is important room for improvement (2, 3). The need for more objective assessment is another reason to avoid surgery and offer TACE to patients who state that their symptoms are severe enough to request intervention.

In the present report, all patients accepted the initial treatment session; 4 patients accepted additional sessions for symptom control. None of the patients with unsatisfactory symptom control wanted to undergo surgery for the residual hemangioma. On a retrospective assessment, initially missed or *de novo* cholecystolithiasis may have been the reason for unsatisfactory symptom control in two patients who were reported as “failures” of TACE. Relief can be derived from the fact that no other significant cause of abdominal pain could be identified during follow up, i.e., a misdiagnosis was excluded. We agree with Furumaya et al. that the schemes we and others have used (4–6) should be replaced by validated quality of life questionnaires (2). The issue of treating enlarging hemangiomas in asymptomatic/mildly symptomatic patients is controversial. Our “working approach” is to consider TACE in progressively enlarging lesions, particularly those larger than 10 cm and abutting major vascular structures (13). However, enlargement should not be an “automatic” indication for intervention. Our experience on 46 adult patients with spontaneously regressing hemangiomas has shown that 15% of the lesions showed enlargement first, followed by regression (22). Except for rapidly enlarging lesions, it may be advisable to document progressive enlargement in two consecutive examinations before planning an intervention.

The patient with fever had a gratifying response to TACE, as reported in abstract form previously (23). The published experience on this rare entity at the time of admission (10/2017) comprised mostly cases treated surgically (24, 25) although patients who recovered with steroids (26) and conservative follow up (27), had been reported. The then 5-year favorable experience at our center, led us to offer TACE first and consider surgery as a second option because there was no evidence of rupture or intra-lesional bleeding. To the best of our information, this is the first reported use of TACE in this condition.

Auto- and allotransplantation are the most extreme surgical interventions for liver hemangiomas. The leading indications are the Kasabach–Merrit phenomenon and the presence of giant hemangioma with hemangiomatosis in the adjacent liver parenchyma (18, 19, 28–30). A frequent feature among the approximately 30 reported patients is that the enlarging hemangioma was followed without effective intervention and eventually the patient was referred to the transplant center. Eight of these patients had undergone embolization (18, 19, 29); two had received polyvinyl alcohol particles and one had received trisacryl gelatin as well; the embolic material was not stated in the remaining 6. Lipiodol-bleomycin use has not been reported. TACE has been used successfully to manage the Kasabach-Merrit phenomenon in an adult (31). The experience reported here on patients with giant hemangiomas with hemangiomatosis in the adjacent parenchyma shows that TACE might have been useful to prevent the need for liver transplantation.

The issue of whether the indications for TACE may be expanded in view of the generally high success and low complications rates (6, 7) and which non-operative intervention is better (32) should be postponed until better assessment of the quality of life in symptomatic patients and a composite definition of success in cases with progressive enlargement are realized.

In conclusion, our hepatopancreatobiliary surgery unit offers TACE, rather than surgery, as a successful first-line treatment for patients with symptomatic/enlarging hemangiomas. A comparative study would have been very valuable for scientific purposes. However, our experience shows that accrual of patients for the surgical arm would be difficult. More data from surgical departments are required to define the respective roles of surgery and embolization in the management of symptomatic/enlarging liver hemangiomas.

## Data Availability

The raw data supporting the conclusions of this article will be made available by the authors, without undue reservation.
